# The Challenge of High Coronary Thrombotic Events in Patients with ST-Segment Elevation Myocardial Infarction and COVID-19

**DOI:** 10.3390/jcm11216542

**Published:** 2022-11-04

**Authors:** Larisa Anghel, Bogdan-Sorin Tudurachi, Andreea Leonte, Radu Andy Sascău, Ioana Mădălina Zota, Amin Bazyani, Grigore Tinică, Cristian Stătescu

**Affiliations:** 1 Internal Medicine Department, “Grigore T. Popa” University of Medicine and Pharmacy, 700503 Iași, Romania; 2 Cardiology Department, Cardiovascular Diseases Institute “Prof. Dr. George I. M. Georgescu”, 700503 Iași, Romania; 3 Interventional Department, Cardiovascular Diseases Institute “Prof. Dr. George I. M. Georgescu”, 700503 Iași, Romania; 4 Cardiovascular Surgery Department, Cardiovascular Diseases Institute “Prof. Dr. George I. M. Georgescu”, 700503 Iași, Romania

**Keywords:** STEMI, COVID-19, SARS-CoV-2, thrombotic events, coronary disease, in-hospital mortality

## Abstract

The aim of this observational study was to describe the characteristics and outcomes of coronavirus disease 2019 (COVID-19)-positive patients with ST-segment elevation myocardial infarction (STEMI), with a special focus on factors associated with a high risk of coronary thrombosis and in-hospital mortality. Comparing the two groups of patients with STEMI separated according to the presence of SARS-CoV-2 infections, it was observed that COVID-19 patients were more likely to present with dyspnea (82.43% vs. 61.41%, *p* = 0.048) and cardiogenic shock (10.52% vs. 5.40%, *p* = 0.012). A longer total ischemia time was observed in COVID-19 patients, and they were twice as likely to undergo coronary angiography more than 12 hours after the onset of symptoms (19.29% vs. 10.13%, *p* = 0.024). In 10 of 57 COVID-19-positive patients, a primary PCI was not necessary, and only thromboaspiration was performed (17.54% vs. 2.70%, *p* < 0.001). Platelet level was inversely correlated (r = −0.512, *p* = 0.025) with a higher risk of coronary thrombosis without an atherosclerotic lesion. Using a cut-off value of 740 ng/ml, D-dimers predicted a higher risk of coronary thrombosis, with a sensitivity of 80% and a specificity of 66% (ROC area under the curve: 0.826, 95% CI: 0.716–0.935, *p* = 0.001). These are novel findings that raise the question of whether more aggressive antithrombotic therapy is necessary for selected COVID-19 and STEMI patients.

## 1. Introduction

Coronavirus disease 2019 (COVID-19) is an important global pandemic with direct and indirect cardiac complications, including acute myocardial infarction (AMI), life-threatening arrhythmias, or heart failure [1]. Also, as a result of coagulation abnormalities, severe acute respiratory syndrome coronavirus 2 (SARS-CoV-2) infection predisposes patients to thrombotic events, both in the arterial and venous circulation [2,3]. Cardiovascular disease is the number one cause of death and human suffering worldwide, accounting for approximately one third of deaths globally [4,5]. Among cardiovascular illnesses, coronary artery disease is acknowledged as the most prevalent, and its incidence is expected to increase as a result of the increasing prevalence of cardiovascular risk factors, and especially because of population aging [2,6]. Impairment of the cardiovascular system is common in patients with SARS-CoV-2 infection, but the exact mechanisms behind it are not fully understood. Acute cardiac injury can be found in approximately 25% of patients with COVID-19 and is associated with a higher risk of mortality, due to late presentation in the hospital and safety measures [5]. Newly emerged data points out that COVID-19 infection, both asymptomatic and symptomatic, is a cardiovascular risk factor that can induce myocardial injury. The exact mechanisms are not fully known, but they may include direct virus invasion, maladaptive host immune responses, microvascular angiopathy, myocardial supply-demand mismatch leading to type II myocardial infarction, takotsubo cardiomyopathy, cytokine storm, and hypercoagulability [4,7]. Particular attention should be given to patients with cancer as that they are in an immunosuppressed state with poor microcirculation, lymphocytopenia, and an alteration of the intestinal bacterial flora. This may be responsible for their high risk of premature cardiovascular diseases and viral infection, leading to more negative prognoses than healthy people. It is known that patients with SARS-CoV-2 infection overproduce pro-inflammatory cytokines, growth factors, and chemokines, which may be involved in myocardial injuries such as myocarditis, acute myocardial infarction, heart failure, and arrhythmia [8]. However, the identification of risk factors for myocardial injury in patients with SARS-CoV-2 is still an area of active investigation, and a more accurate understanding of them will help to improve clinical outcomes.

Despite all the efforts made by the international and national health authorities to fight the SARS-CoV-2 infection, the impact on the cardiovascular system still remains very important. Also, data regarding the clinical and paraclinical presentation, treatment, outcomes, and in-hospital mortality of patients with AMI and COVID-19 are still a matter of debate.

The aim of this study is to provide a complete insight into the demographic characteristics, management strategies, and outcomes of COVID-19 patients with ST-segment elevation myocardial infarction (STEMI) from a primary percutaneous coronary intervention (PCI) center in the north-east of Romania, with a special focus on factors associated with a high risk of coronary thrombosis and in-hospital mortality.

## 2. Materials and Methods

### 2.1. Study Design and Patient Population

This is a prospective observational study conducted on STEMI patients admitted to the Intensive Cardiac Care Unit of the “Prof. Dr. George I.M. Georgescu” Cardiovascular Disease Institute, Iasi, Romania, during the COVID-19 period, from 1 March 2020 (date of first COVID-19 case in the hospital) through 1 May 2022. To overcome potential preselection bias, there was no significant difference between groups regarding age, gender, presence of associated comorbidities, or location of the myocardial infarction. A total of 2341 patients with STEMI were admitted to our primary percutaneous coronary intervention center from 1 March 2020 to 1 May 2022. Of these, 57 patients had known or confirmed COVID-19 infection upon admission. They were compared with 74 matched control patients without COVID-19 infection, treated in the same period. We compared in-hospital mortality and complications between the groups of COVID-19 and non-COVID-19 patients with STEMI and evaluated whether the presence of COVID-19 was associated with a high risk of coronary thrombosis without coronary plaque disruption, and in-hospital mortality. The study was performed in accordance with the Declaration of Helsinki, and its subsequent amendments were conducted as requested by the hospital Ethical Committee. All data were collected anonymously, and patients routinely subscribed to a disclosure statement for the use of personal data at the beginning of hospitalization.

### 2.2. Data Collection and Definitions

Demographic information, personal and familial history, therapeutic management, and in-hospital outcomes were recorded, including age, sex, body mass index, smoking, dyslipidemia, hypertension, diabetes mellitus, and prior coronary artery disease or heart failure. For each STEMI patient, the time from symptom onset to hospital admission was recorded, along with the time from hospital admission to reperfusion. The symptom-onset time was documented as the moment when the patient reported cardiovascular symptoms, such as chest pain, dyspnea, syncope, or palpitations, but not respiratory symptoms. Data regarding clinical manifestations, electrocardiographic and echocardiographic findings, as well as the results of laboratory tests and procedure-related findings, were recorded. Thus, we assessed data regarding cardiac troponin, creatine kinase, complete blood count, fibrinogen, C-reactive protein, D-dimer, ferritin, and kidney and liver function. None of the patients included in the study had a history of thrombophilia or other previous thrombotic events.

STEMI was defined based on the presence of typical symptoms associated with ST-segment elevation, more than 1 mm in two consecutive leads, or a new left bundle-branch block, with or without elevated cardiac markers.

Patients were classified according to their COVID-19 status into two groups: patients with STEMI and COVID-19 infection, and patients with STEMI without COVID-19 infection. In each patient, COVID-19 infection was confirmed with reverse transcription-polymerase chain reaction assays from throat swabs or lower respiratory tract samples. These patients were managed as COVID-19 positive, as per center policy.

The culprit lesion was considered in case of angiographic evidence of thrombotic occlusion/subocclusion. The number of coronary vessels with severe stenosis (angiographic evidence of a stenosis > 70% on visual estimation), number of stents, and angiographic characteristics were also assessed.

The following in-hospital complications were also recorded: ventricular arrhythmia, atrial fibrillation, second- or third-degree atrioventricular block, mechanical ventilation, and in-hospital mortality.

### 2.3. Statistical Analysis

Data were analyzed using the Statistical Package for the Social Sciences v.26 (SPSS, Chicago, IL, USA). The Shapiro–Wilk test was used for checking the distribution of the continuous variables. The continuous variables were compared using an independent two-sample *t*-test where there was a normal distribution of data, or the Mann–Whitney U test in cases of the non-normal distribution of data. Continuous numerical variables which had normal distributions were summarized as mean ± standard deviation, while variables with non-normal distributions were expressed as median and interquartile ranges (IQRs). A chi-square test was used to compare the categorical variables, which are presented as frequencies and percentages. We employed a Pearson correlation test to evaluate the potential associations between the presence of cardiovascular risk factors and the severity of coronary lesions, and also between inflammatory markers and the presence of acute coronary atherothrombosis in COVID-19-positive patients. The results are reported with regard to COVID-19 diagnosis and compared with control patients. Receiver operating characteristic (ROC) curve analysis was conducted to assess the optimum cut-off value of D-dimers in predicting a higher risk of coronary thrombosis for patients with myocardial infarction and SARS-CoV-2 infection. A *p*-value < 0.05 was considered statistically significant for all analyses.

## 3. Results

A total of 2341 patients with STEMI were admitted to the Intensive Cardiac Care Unit of the “Prof. Dr. George I.M. Georgescu” Cardiovascular Disease Institute, Iasi, from 1 March 2020 to 1 May 2022. Of these, 57 patients had known or confirmed SARS-CoV-2 infection upon admission. They were compared with 74 matched control patients without COVID-19 infection who were treated in the same period.

### 3.1. Baseline Characteristics

Patients included in the study were typically male (86.25%), from urban areas (59.54%), between 40 and 89 years of age, and there were no statistically significant differences between groups.

Chest pain was the most common presenting symptom in both groups, but a significant proportion of patients with COVID-19 presented with dyspnea compared to patients in the control group (82.43% vs. 61.41%, *p* = 0.048). Also, a statistically significant difference was observed between the two groups in terms of oxygen saturation at admission. Patients with SARS-CoV-2 infection had lower saturation values at admission (92% vs. 97%, *p* = 0.018), with five patients requiring orotracheal intubation and mechanical ventilation since admission, compared to only two patients in the control group. During hospitalization, two other patients with COVID-19 required orotracheal intubation and assisted mechanical ventilation, before having a coronarography performed, while only one patient in the control group required noninvasive ventilatory support. The two patients with COVID-19 required orotracheal intubation in the context of acute pulmonary edema, which was unresponsive to standard medical treatment. Considering the fact that in our clinic we treat only patients with cardiovascular disease, we are not able to provide long-term care for those with SARS-CoV-2 infection. Thus, we were unable to perform lung computed tomography scans for all these patients to assess lung damage, but all of them had chest X-rays, and most had a mild or moderate form of SARS-CoV-2 infection.

Concerning concomitant and previous illnesses, there was no significant difference between groups regarding the presence of arterial hypertension, diabetes mellitus, smoking status, dyslipidemia, chronic kidney disease, or location of myocardial infarction. Instead, patients in the control group more often had a history of coronary artery disease, both myocardial infarction and interventional and surgical myocardial revascularization.

Regarding clinical assessment, most patients presented with KILLIP I or KILLIP II heart failure, but a significant proportion of patients with SARS-CoV-2 infection had high-risk pre-PCI conditions, such as cardiogenic shock (10.52% vs. 5.40%, *p* = 0.012).

All patients included in the study were referred to our primary percutaneous coronary intervention center for angiographic evaluation. Thus, all COVID-19-positive patients and those from the control group underwent angiography. We observed a longer total ischemia time in COVID-19-positive patients relative to the control group. Thus, patients with SARS-CoV-2 infection were almost twice as likely to undergo coronary angiography more than 12 h after the onset of symptoms, as compared to the control group (19.29% vs. 9.46%, *p* = 0.024). Baseline demographic and clinical characteristics are presented in Table 1.

### 3.2. Paraclinical Characteristics

#### 3.2.1. Laboratory Findings

Significantly, higher values of inflammatory biomarkers, such as white blood cells (12,546 ± 1077 vs. 10,655 ± 988, *p* = 0.001), C-reactive protein (CRP) (92.64 ± 13.36 vs. 74.36 ± 12.01, *p* = 0.002), erythrocyte sedimentation rate (ESR) (42.74 ± 4.03 vs. 33.89 ± 10.23, *p* = 0.001), and serum ferritin (453 ± 87.13 vs. 389 ± 27.44, *p* = 0.025) were observed in COVID-19-positive patients. Concerning the coagulation parameters, fibrinogen and D-dimer values were also significantly higher in COVID-19-positive patients. There was a trend towards an increased value of troponin and lactate dehydrogenase (LDH) in patients with SARS-CoV-2 infection, probably in the context of later presentation, and, statistically, this was significant. In terms of lipid profile values, patients in the control group had higher values of low-density lipoprotein (LDL) cholesterol, but this was not statistically significant (105 ± 50.98 vs. 111 ± 47.36, *p* = 0.227). Laboratory findings for the patients included in the study are presented in Table 2.

#### 3.2.2. Echocardiographic and Procedural Characteristics

Concerning the echocardiographic characteristics, we observed a statistically significant difference in terms of left ventricular ejection fraction values, with ventricular dysfunction being more severe in the control group (39 (32–56) vs. 33 (30–48), *p* = 0.044).

All patients included in the study underwent coronary angiography. We observed a significant difference in the time of total ischemia, with COVID-19-positive patients having longer total ischemia times. Most of the patients included in the study underwent coronary angiography between 6 and 12 h after the onset of symptoms, and only a small number in the first 6 h after the onset. Of the 57 COVID-19-positive patients, 9 patients had no atherosclerotic lesions compared to only 1 patient in the control group (15.78% vs. 1.35%, *p* = 0.020). Patients without atherosclerotic lesions received only one antiplatelet drug along with unfractionated heparin during hospitalization, and novel oral anticoagulant treatment at discharge for at least 30 days, also according to the recommendations of the infectionist. Almost half of the patients in both groups had unicoronary lesions, and no significant difference was observed in culprit vessels, lesion location, or multivessel disease. There were no complications during the coronarographies, and the final coronary flow was reported as TIMI 2 or 3. In 10 of the 57 COVID-19-positive patients, only thromboaspiration was performed (17.54% vs. 2.70%, *p* < 0.001) and a primary PCI was not necessary. In contrast, patients from control group were more likely to receive coronary angioplasty and less likely to receive medical management alone, without reperfusion. Regarding other reperfusion strategies, only one patient from the control group received thrombolytic treatment with reteplase, but without reperfusion criteria, and a rescue PCI was performed. None of the patients included in the study required emergency coronary artery bypass grafting (Table 3).

The Pearson’s correlation was used in order to see if there was a linear relationship between cardiovascular risk factors and the severity of coronary lesions in COVID-19-positive patients. There was a significant positive correlation between active smoker status and the severity of coronary lesions (r = 0.856. *p* = 0.008). Concerning the other cardiovascular risk factors, a moderate correlation was observed only for diabetes mellitus (r = 0.656. *p* = 0.035) and dyslipidemia (r = 0.565. *p* = 0.048) (Table 4).

### 3.3. Correlations between Biological Markers and the Presence of Coronary Thrombosis in COVID-19-Positive Patients

**Figure 1:**
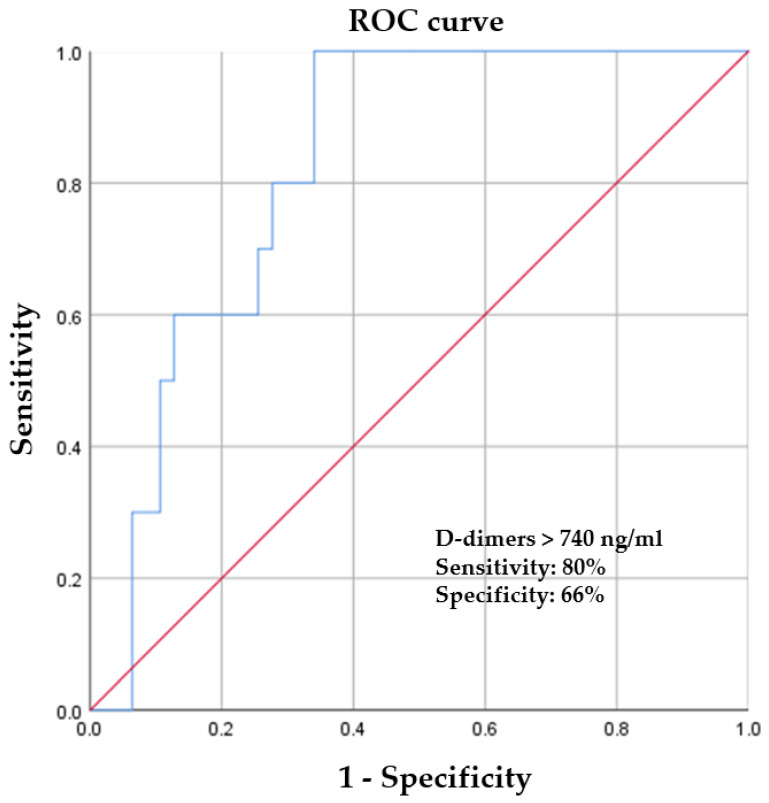
Receiver operating characteristic curve of D-dimers in predicting a higher risk of coronary thrombosis for patients with myocardial infarction and SARS-CoV-2 infection.

During the study period, we observed that a high number of COVID-19-positive patients had no atherosclerotic lesions, and a primary PCI was not necessary. Instead, only thromboaspiration was performed. Thus, through studying the correlation between prothrombotic markers and the presence of coronary thrombosis in COVID-19-positive patients, we observed that the D-dimer level was positively correlated (r = 0.786, *p* = 0.006) and platelets were inversely correlated (r = −0.512, *p* = 0.025) with a higher risk of coronary thrombosis, without an atherosclerotic lesion. D-dimers predicted a higher risk of coronary thrombosis (with a cut-off value of 740 ng/mL), with a sensitivity of 80% and a specificity of 66% (ROC area under the curve: 0.826, 95% CI: 0.716 to 0.935, *p* = 0.001), for patients with myocardial infarction and SARS-CoV2 infection (Figure 1).

Also, there was a significant positive correlation between high values of inflammatory markers, such as CRP, fibrinogen, and ESR, and the presence of acute coronary atherothrombosis (r = 0.703, *p* = 0.005, in the case of CRP, and r = 0.756, *p* = 0.001 for fibrinogen) (Table 5).

### 3.4. In-Hospital Outcome

A significantly higher in-hospital mortality was observed among patients positive for COVID-19. In fact, 9 of the 57 positive patients died, compared to only 1 patient in the control group (15.78% vs. 1.35%, *p* < 0.001). Two patients with COVID-19 infection died due to ventricular arrhythmia, two from cardiogenic shock, and two from septic shock, with electromechanical dissociation unresponsive to cardiopulmonary resuscitation, and in three cases, cardiac rupture occurred as a mechanical complication of acute myocardial infarction. In comparison, the patient in the control group died secondary to cardiac rupture in the 4th day after the onset of the acute coronary event. We consider that the increased number of patients with cardiac rupture may be secondary to the late revascularization of these patients. Regarding other complications, ventricular arrhythmia was more frequent in patients with SARS-CoV-2 infection, and atrial fibrillation was more common in patients from the control group (Table 6).

Patients from the control group had longer lengths of hospital stay and intensive care unit stay.

## 4. Discussion

In our study, we were able to assess all 57 STEMI patients confirmed to have COVID-19 who had been hospitalized in a primary PCI center in the north-east of Romania between March 2020 and May 2022. They were compared with 74 matched control patients without COVID-19 infection, treated in the same period.

**Figure 2:**
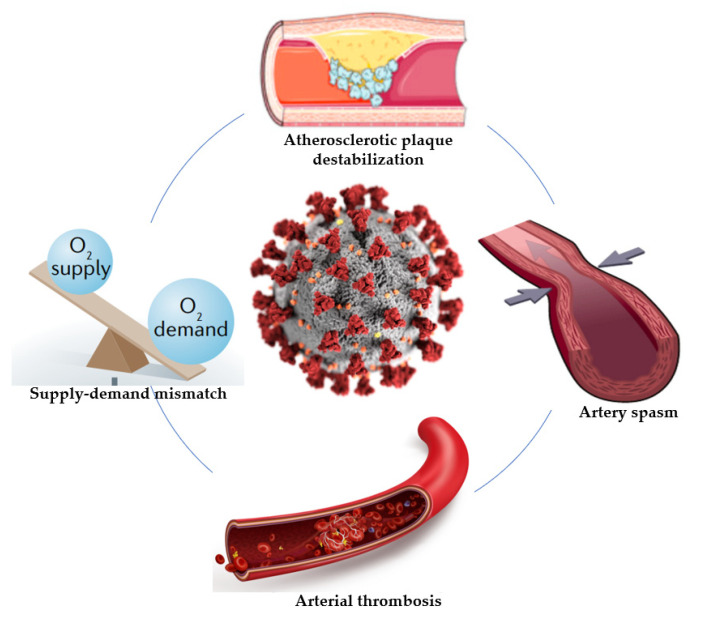
Possible mechanisms associated with myocardial infarction in patients with COVID-19.

There are several important findings from this study. First, COVID-19 patients were more likely to present with dyspnea and high-risk pre-PCI conditions, such as cardiogenic shock. Second, a longer total ischemia time was observed in patients with SARS-CoV-2 infection, and they were almost twice as likely to undergo coronary angiography more than 12 h after the onset of symptoms, despite these high-risk pre-PCI conditions. Third, significantly higher values of inflammatory and coagulation biomarkers were observed in COVID-19-positive patients, though left ventricular systolic dysfunction was more severe in the control group. Fourth, in almost 20% of COVID-19-positive patients, a primary PCI was not necessary, and only thromboaspiration was performed. A significant positive correlation was observed between active smoker status and the severity of coronary lesions in COVID-19-positive patients. Therefore, there are many mechanisms responsible for the occurrence of myocardial infarction in patients with COVID-19, and understanding this relationship is very important in order to prevent an increase in mortality and the risk of complications (Figure 2).

Since the beginning of the COVID-19 pandemic, many studies have focused on pulmonary findings in SARS-CoV-2 infection, and have evaluated the consequences of infection of the heart. All of these studies have emphasized that patients with cardiovascular diseases were at higher risk of developing complications, further cardiac events, and severe forms of COVID-19, and were at higher risk of mortality as well [9,10,11,12,13,14,15]. 

The COVID-19 pandemic is a dynamic phenomenon, and the impact has varied worldwide. In general, in our country, as in other countries, in the first months of the pandemic, the health system was overwhelmed, and it was unable to deliver timely and effective treatment. Also, a decline in myocardial infarction hospitalizations was observed, which can be explained in the context of that difficult period both by patients’ fear of being infected with SARS-CoV-2, and also their desire not to overwhelm the medical system. Thus, even if these consequences of the COVID-19 pandemic were observed worldwide, the results of our study reflect the impact of the pandemic only on patients in this area of the country, and they should not be generalized.

Regarding the clinical characteristics of AMI patients and SARS-CoV-2 infection, we observed that, during the study period, patients with STEMI hospitalized in our clinic with SARS-CoV-2 infection were typically male and had a mean age of 64 years old. Our findings are consistent with the results observed in other studies that evaluated clinical data about patients with STEMI and SARS-CoV-2 infection [14,15,16,17]. For example, data from the North American COVID-19 Myocardial Infarction (NACMI) Registry show that male gender and advanced age were risk factors for CVD in patients with COVID [10].

An important observation common in previous studies was that patients with SARS-CoV-2 infection and STEMI presented more frequently with dyspnea and atypical symptoms [18,19,20,21]. Atypical presentation of STEMI or these overlapped respiratory symptoms might explain the decrease in the willingness of patients to seek medical care and also their late presentation, with important complications such as cardiogenic shock or the need for orotracheal intubation and mechanical ventilation [17,20,22,23,24,25,26,27]. In line with these observations, the results from the North American COVID-19 Myocardial Infarction Registry (NACMI), an ongoing observational registry, demonstrated that patients with STEMI and COVID-19 presented more frequently with high-risk features, such as shock and dyspnea [10]. It is difficult to determine whether these symptoms are due to COVID-19 specifically or are a consequence of cardiac involvement, or both, considering the endothelial dysfunction, increased cytokine level, oxidative stress, and low oxygen saturation that lead to ischemia and low cardiac output. We also observed a significant proportion of patients with SARS-CoV-2 infection admitted with high-risk pre-PCI conditions. Thus, cardiogenic shock was more frequent in patients with COVID-19 (10.52% vs. 5.40%, *p* = 0.012), and five patients required orotracheal intubation and mechanical ventilation since admission, compared to only two patients in the control group.

Concerning procedural characteristics, most previous studies have observed longer total ischemia time and time from the first medical contact to coronary angiography during this pandemic period [28,29]. Also, regardless of the reason for the delay, some studies have reported a significant increase in the risk of death and adverse events [13,28,30]. All patients included in our study underwent coronary angiography, but patients with SARS-CoV-2 infection were almost twice as likely to undergo coronary angiography more than 12 h after the onset of symptoms, compared to the control group. This may be one of the reasons for the increased risk of death and adverse events which we observed in our study, since each 30 min delay to primary percutaneous angioplasty increases the risk for in-hospital mortality by up to 20–30% [26,27,31]. Thus, it is very important to take all possible efforts to minimize the total myocardial ischemia time and provide optimal healthcare services for STEMI patients in this pandemic period.

To date, several possible mechanisms linking SARS-CoV-2 infection to acute myocardial infarction have been advocated. One potential mechanism is the endothelial injury induced by SARS-CoV-2 infection, which may further induce coronary thrombosis [7,8]. The pro-inflammatory state present in patients with COVID-19 may promote the destabilization of coronary atherosclerotic plaques, with increasing risk of plaque rupture and coronary thrombosis [4,6]. In our study, we observed a significant positive correlation between high values of CRP, fibrinogen, and ESR and the presence of acute coronary atherothrombosis. Moreover, low platelet counts and high D-dimer levels were associated with a higher risk of thrombosis, but without an underlying atherosclerotic plaque. This low platelet count was often described in patients with SARS-CoV-2 infection and may suggest an increased consumption as a result of platelet activation and thrombus formation [2,8,32,33,34]. Another possible mechanism responsible for the appearance of type 2 AMI in these patients is the mismatch between myocardial oxygen supply and demand. Thus, due to sympathetic system activation and hypoxemia in the context of acute respiratory insufficiency, patients with COVID-19 have an increased myocardial oxygen demand and a reduced oxygen supply [6]. Also, the occurrence of myocardial infarction in patients with SARS-CoV-2 infection may be related to endothelial and microvascular injuries, which can lead to inflammation, thrombosis, or coronary vasospasm [7,8]. The pro-inflammatory state associated with SARS-CoV-2 infection may also activate inflammatory cells from the atherosclerotic plaques, increasing their vulnerability and the risk of complications, such as coronary thrombosis [6]. Canzano et al. revealed that the cytokine storm and the imbalance of the endothelial functions present in COVID-19 patients may be involved in the appearance of microthrombi. Also, their results provide insights into the IL-6–mediated platelet activation that may be responsible for small local thrombi, despite anticoagulant treatment, suggesting the potential effectiveness of anti–IL-6 antibodies and antiplatelet drugs in such cases [7]. The cytokine storm has a central role in the prognosis of patients with SARS-CoV-2 infection. Also, the increased level of D-dimer found in 90% of patients is another independent risk factor for cardiovascular mortality. All these factors contribute to the appearance and progression of endothelial damage, stimulating the release of pro-coagulant mediators involved in thrombosis and ischemia. Therefore, it is speculated that cytokine-related pathways should be studied and promoted as potential targets to improve the outcomes of patients with COVID-19 [7,8]. Currently available data suggests some innovative therapies aimed to reduce mortality in patients with STEMI and SARS-CoV-2 infection, including selective inhibitors of interleukin 1 or interleukin 6, especially in vulnerable patients, such as those with cancer. Tocilizumab, an antibody that competitively inhibits the binding of IL-6 to its receptor (IL-6R), is already used by oncologists to reduce pulmonary distress in cancer patients during immune checkpoint inhibitors. It binds soluble as well as membrane-bound interleukin-6 receptors, stopping IL-6 from exerting its pro-inflammatory effects. There are ongoing studies related to canakinumab, an antibody against IL-1 beta which is known for its cardioprotective effects in high-risk patients and has no cross-reactivity with other members of the interleukin-1 family, such as interleukin-1 alpha [35]. However, further studies are required to reveal the underlying mechanism of coronary thrombosis in patients with STEMI and SARS-CoV-2 infection.

To our knowledge, this study adds important insights regarding the correlation between inflammatory and prothrombotic markers and the presence of coronary thrombosis in COVID-19-positive patients. There were a significant positive correlation between high values of inflammatory markers, such as CRP, fibrinogen, and ESR, and the presence of acute coronary atherothrombosis. Also, high levels of D-dimers and low platelet counts were associated with a higher risk of thrombosis, without an atherosclerotic lesion.

Pellegrini et al. conducted a systematic pathological analysis of 40 hearts from hospitalized patients dying of COVID-19 in Bergamo, Italy, in order to study the mechanism and the type of myocardial damage associated with SARS-CoV-2 infection. Cardiac thrombi were present in 78.6% cases with myocardial necrosis, most of them being present in myocardial capillaries, arterioles, and small muscular arteries. Compared with epicardial coronary thrombus aspirates from STEMI cases, these microthrombi had a different composition, consisting of higher levels of terminal complement and fibrin [9]. These results may have important clinical implications, such as the possibility of using tailored antithrombotic strategies to counteract the cardiac effects of SARS-CoV-2 infection. Moreover, in experimental models, inhibiting the complement pathway seems to have a possible therapeutic effect [36]. In another study, Bois et al. reported nonocclusive microthrombi in the small intramyocardial vasculature in 80% of COVID-19 autopsy cases, and only 2 out of 15 patients had acute ischemic injury [37]. Furthermore, in a case series involving 18 patients with STEMI and COVID-19, 56% had nonobstructive disease on coronary angiography, and they had a high mortality rate (90% died in the hospital) [38].

In this context, assessing the etiology of myocardial injury in patients with SARS-CoV-2 may be a real challenge in current clinical practice, due to the possibility of microthrombi, which may not be detectable clinically. It is also very important to find different methods and laboratory tests that can specifically detect cardiac microthrombi responsible for myocardial injury in patients with COVID-19. In future study, there is a desperate need for better understanding of the pathogenesis of myocardial injury in patients with SARS-CoV-2 infection, and to find the best way to treat them.

Median peak plasma hs-troponin concentration was greater in the COVID-19 group compared with the non-COVID-19 group, but without statistically significant differences. What is interesting is that the left ventricular ejection fraction was significantly lower in the non-COVID-19 group (32.91 ± 3.67 vs. 38.02 ± 3.12, *p* = 0.044). This may be due to the fact that the hs-troponin dosing was performed only once, at admission, and also due to the heterogeneity of the control population. In the future, we intend to try to reduce this bias by performing more accurate propensity matching using many variables.

The results from the ongoing observational North American COVID-19 Myocardial Infarction Registry demonstrated that patients with STEMI and COVID-19 had worse prognoses, longer hospital courses, and significantly higher mortality rates [10]. Comparing admission rate, hospitalization course, and mortality of patients hospitalized with acute myocardial infarction in England during the COVID-19 period, Rashid et al. [39] demonstrated higher early mortality from acute myocardial infarction during the COVID-19 pandemic compared with before. Along these lines, we observed higher early mortality rates in COVID-19-positive patients with STEMI (15.78% vs. 1.35%, *p* < 0.001). Also, these patients had higher risk of other complications, such as ventricular arrhythmia. In contrast, patients from the control group had longer hospital stays. This difference is most likely due to the fact that our clinic does not have many beds dedicated to the care of patients with SARS-CoV-2 infection, and most positive patients are subsequently sent to COVID-19 support hospitals, in order to continue post-angioplasty monitoring. All these results indicate that an optimization of care is necessary regarding the management of patients with STEMI and SARS-CoV-2 infection.

There are multiple strengths and limitations to our study. The current paper adds important insights regarding the correlation between high values of CRP, fibrinogen, and ESR and the presence of acute coronary atherothrombosis in patients with COVID-19. Also, the association of low platelet counts and high D-dimer levels with an increased risk of coronary thrombosis, without an underlying atherosclerotic plaque, is another novelty. Despite these multiple strengths, there are also some limitations. One of the most important limitations of our study is the heterogeneity of the population included. We intend to continue the study and to do a long-term follow-up of these patients, and we will perform a propensity matching with many variables in order to reduce this bias. This is a relatively small observational study in a single center, and thus has all the limitations of this type of analysis, such as bias and the potential for confounding. Due to the study’s nature and design, the follow-up duration was short, and involved only in-hospital stay. Unfortunately, considering the epidemiological context in which we conducted this study, it was very difficult to evaluate the patients after discharge from our hospital. Thus, the impossibility of short- and long-term monitoring of these patients is another limitation of the study. The long-term observation of patients might be crucial for better assessment of the implications of COVID-19 in patients with STEMI, especially in those without atherosclerotic coronary plaque, and we intent to evaluate these patients at a mean period of 12 months after the acute coronary event. Also, this is a small monocentric study, and the results reflect the impact of the pandemic only on patients in this area of the country, and they should not be generalized.

## 5. Conclusions

This is one of the first comparative studies from our country of COVID-19-positive versus non-COVID-19 patients presenting with ST-segment elevation myocardial infarction. We observed a significant positive correlation between high values of CRP, fibrinogen, and ESR and the presence of acute coronary atherothrombosis. The strong association between low platelet counts and high D-dimer levels with a higher risk of coronary thrombosis, without an atherosclerotic plaque, raises the question of whether more aggressive antithrombotic strategies might be used to counteract the effects of microthrombi on the heart in selected COVID-19 and STEMI patients. We, therefore, highlight that this is only a small monocentric study, with a special focus on factors associated with a high risk of coronary thrombosis and in-hospital mortality in patients with STEMI and COVID-19, and further investigation on this topic should be conducted in clinical trials.

## Figures and Tables

**Table 1:** Overview of baseline demographic and clinical characteristics.

Variable	COVID + Patients (n = 57 Patients)	Control Patients (n = 74 Patients)	*p* Value
**Baseline characteristics**			
**Age (years)**	64 (40–89)	64 (41–88)	0.860
**Male**	49 (85.96%)	64 (86.48%)	0.564
**Urban area**	34 (59.64%)	44 (59.45%)	0.563
**Admission complaints**			
**Chest pain**	43 (75.43%)	57 (77.02%)	0.496
**Dyspnea**	47 (82.43%)	46 (61.41%)	0.048
**Medical history**			
**Prior MI**	9 (15.78%)	25 (33.78%)	0.016
**Prior PCI**	6 (10.52%)	11 (14.86%)	0.595
**Prior CABG**	2 (3.51%)	3 (4.05%)	0.622
**Risk factors**			
**Diabetes mellitus**	30 (52.70%)	33 (44.59%)	0.567
**Hypertension**	32 (56.14%)	44 (59.45%)	0.419
**Obesity**	29 (50.87%)	34 (45.94%)	0.289
**BMI (kg/m^2^)**			
**<18**	6 (10.52%)	7 (9.45%)	
**18–30**	22 (38.59%)	33 (44.59%)	
**30–40**	25 (43.85%)	31 (41.80%)	
**>40**	4 (7.14%)	3 (4.05%)	
**Dyslipidemia**	19 (33.33%)	25 (33.78%)	0.476
**Chronic kidney disease**	2 (3.50%) (≥G3a)	2 (2.70%) (≥G3a)	0.129
**Active smoker**	16 (28.07%)	20 (27.02%)	0.872
**Admission hemodynamics**			
**SBP (mmHg)**	132 (88–145)	145 (120–174)	NA
**HR (bpm)**	67 (35–87)	78 (56–112)	NA
**Oxygen saturation aa (%)**	92% (81–100)	97% (92–100)	0.018
**Total ischemia time**			
**<6 h**	5 (8.77%)	13 (17.56%)	0.016
**6–12 h**	41 (71.94%)	54 (72.98%)	0.563
**>12 h**	11 (19.29%)	7 (9.46%)	0.024
**Killip Class**			
**I**	46 (80.71%)	66 (89.19%)	0.187
**II**	3 (5.26%)	4 (5.40%)	0.406
**III**	2 (3.51%)	1 (1.36%)	0.281
**IV**	6 (10.52%)	3 (4.05%)	0.012

aa, atmospheric air; BMI, body mass index; CABG, coronary artery bypass graft surgery; HR, heart rate; MI, myocardial infarction; PCI, percutaneous coronary intervention; SBP, systolic blood pressure.

**Table 2:** Laboratory findings for patients included in the study.

Variable	COVID + Patients (n = 57 Patients)	Control Patients (n = 74 Patients)	*p* Value
**Hemoglobin (g/dL)**	13.5 (12.5–14.6)	13.8 (12.8–14.8)	0.565
**Hematocrit (%)**	41.8 (39.5–44.8)	42.3 (38.8–44.6)	0.239
**Platelets (mm^3^)**	270,000 (210,000–450,000)	265,000 (190,000–345,000)	0.805
**White blood cells (mm^3^)**	12,500 (8800–14,500)	10,600 (9000–11,500)	0.001
**LDL cholesterol (mg/dL)**	105 (78–180)	111 (88–130)	0.227
** Creatinine (mg/dL)**	1.06 (0.9–1.78)	1.04 (0.8–1.98)	0.636
** Uric acid (mg/dL)**	7.6 (7.3–8.0)	7.2 (6.8–7.9)	0.185
** ALAT (U/L)**	84 (45–110)	82 (38–138)	0.846
** ASAT (U/L)**	122 (100–168)	117 (98–156)	0.688
** CK-MB (U/L)**	156 (55–168)	118 (88–256)	0.061
** LDH (U/L)**	1215 (868–2300)	872 (456–1300)	0.016
** hs-cTnI (ng/L)**	2470 (1800–3400)	1218 (654–1560)	0.001
** CRP (mg/L)**	93 (40–128)	65 (48–82)	0.002
** ESR (mm/1h)**	58 (42–88)	33 (22–55)	0.001
** Fibrinogen (mg/dL)**	640 (560–780)	450 (390–520)	0.023
** Ferritin (ng/mL)**	480 (390–620)	350 (290–410)	0.025
** D-dimer (ng/mL)**	1450 (1100–1780)	490 (380–580)	0.007

ALAT, alanine aminotransferase; ASAT, aspartate aminotransferase; CK-MB, creatine kinase-MB; CRP, C-reactive protein; ESR, erythrocyte sedimentation rate; hs-cTnI, high sensitivity cardiac troponin I; LDH, lactate dehydrogenase; LDL, low-density lipoprotein.

**Table 3:** Echocardiographic and procedural characteristics.

Variable	COVID + Patients (n = 57 Patients)	Control Patients (n = 74 Patients)	*p* Value
**Echocardiographic characteristics**			
**Mean LVEF (%)**	39 (32–56)	33 (30–48)	0.044
**Number of coronary lesions**			
**Without atherosclerotic lesions**	9 (15.78%)	1 (1.35%)	0.020
** Unicoronary lesion**	31 (54.38%)	39 (52.70%)	0.423
** Bicoronary lesions**	8 (14.03%)	18 (24.32%)	0.068
** Tricoronary lesions**	9 (15.78%)	16 (21.62%)	0.085
**Primary PCI**			
** LAD**	23 (40.35%)	34 (45.94%)	0.134
** RCA**	7 (12.28%)	8 (10.81%)	0.204
** LCX**	14 (24.56%)	24 (32.43%)	0.098
** Thromboaspiration (no need for primary PCI)**	10 (17.54%)	2 (2.70%)	<0.001

LAD, left anterior descending artery; LCX, left circumflex artery; LVEF, left ventricular ejection fraction; RCA, right coronary artery; PCI, percutaneous coronary intervention.

**Table 4:** Correlations between the presence of cardiovascular risk factors and the severity of coronary lesions in COVID-19-positive patients.

Risk Factor	Pearson r Correlation Coefficient	*p* Value
**Diabetes mellitus**	0.656	0.035
**Hypertension**	0.111	0.211
**Obesity**	0.133	0.130
**Dyslipidemia**	0.565	0.048
**Chronic kidney disease**	0.162	0.065
**Active smoker**	0.856	0.008
**Alcohol consumption**	0.056	0.524

**Table 5:** Correlations between inflammatory markers and the presence of acute coronary atherothrombosis in COVID-19-positive patients.

Inflammatory Marker	Pearson r Correlation Coefficient	*p* Value
**CRP (mg/L)**	0.703	0.005
**ESR (mm/1h)**	0.686	0.018
**Fibrinogen (mg/dL)**	0.756	0.001
**WBC (mm^3^)**	0.173	0.199
**Ferritin (ng/mL)**	0.099	0.465

CRP, C-reactive protein; ESR, erythrocyte sedimentation rate; WBC, white blood cells.

**Table 6:** In-hospital outcomes and length of hospital stay.

Variable	COVID + Patients (n = 57 Patients)	Control Patients (n = 74 Patients)	*p* Value
**Ventricular tachycardia/fibrillation**	3 (5.26%)	2 (2.70%)	0.044
**Atrial fibrillation**	3 (5.26%)	8 (10.81%)	0.012
**Mean length of hospital stay (days)**	4 (1–8)	6 (3–9)	0.029
**In-hospital mortality**	9 (15.78%)	1 (1.35%)	<0.001

## Data Availability

The data presented in this study are available on request from the corresponding author.
